# Supplementary material for: The initial stages of cement hydration at the molecular level
Source: Nat Commun. 2024 Mar 29;15:2731. doi: 10.1038/s41467-024-46962-w (PMC10980771; doi:10.1038/s41467-024-46962-w)
Supplement: Supplementary file 1 — Supplementary Information [file 41467_2024_46962_MOESM1_ESM.pdf]

Supplementary Information

The initial stages of cement hydration at the molecular level

Xinhang Xu[1], Chongchong Qi[1,2,3*], Xabier M. Aretxabaleta[4], Chundi Ma[1], Dino Spagnoli[2], Hegoi Manzano[4]

Author affiliations

[1] School of Resources and Safety Engineering, Central South University, Changsha 410083, China

[2] School of Molecular Sciences, University of Western Australia, Perth, 6009, Australia

[3] School of Metallurgy and Environment, Central South University, Changsha 410083, China

[4] Department of Physics, Faculty of Science and Technology, University of the Basque Country UPV/EHU, Barrio Sarriena s/n, 48940 Leioa, Bizkaia, Spain

* Corresponding author. Email: chongchong.qi@csu.edu.cn

This file includes:

Supplementary Tables 1 – 4

Supplementary Figures 1 – 14

Supplementary Notes 1 – 4

Supplementary Table 1. The number of Ca-O_w and O_w-H_{ab} bonds of β-C_2S (100) and M3-C_3S (010).

Type	Time (ns)	Counts		Ratios of Ca-O_w bonds and O_w-H_{ab} bonds
		Ca-O_w bonds	O_w-H_{ab} bonds	
β-C_2S (100)	1.5	129	153	1.19
	6	148	189	1.28
	1.5–6	19 (Increased)	36 (Increased)	1.89
M3-C_3S (010)	1.5	193	201	1.04
	6	225	233	1.04
	1.5–6	32 (Increased)	32 (Increased)	1

Supplementary Table 2. The structures of six-coordinated Ca ions of β-C_2S (100) between 1.5 and 6 ns

Structures*	Counts	Structures*	Counts
(4, 1, 1)	25337	(2, 2, 2)	159
(4, 0, 2)	5441	(2, 3, 1)	0
(4, 2, 0)	3012	(2, 4, 0)	0
(3, 1, 2)	11374	(1, 0, 5)	0
(3, 0, 3)	19538	(1, 1, 4)	2502
(3, 2, 1)	179	(1, 2, 3)	1375
(3, 3, 0)	0	(1, 3, 2)	0
(2, 1, 3)	33137	(1, 4, 1)	0
(2, 0, 4)	10272	(1, 5, 0)	0

* The structures of Ca ions are presented in parentheses as the number of Ca-O_s bonds, the number of ligand teeth, and the number of Ca-H_2O_w bonds.

Supplementary Table 3. The coordination numbers and structures of the Ca ions in the transition step of β-C_2S (100).

Coordination number	Structures*	Counts	Percentage
5	(5, 0, 0)	654717	8.760%
6	(5, 1, 0)	847020	11.333%
	(5, 0, 1)	877312	11.738%
7	(5, 2, 0)	1197823	16.027%
	(5, 1, 1)	3047197	40.771%
	(5, 0, 2)	619071	8.283%
8	(5, 3, 0)	603	0.008%
	(5, 2, 1)	45330	0.607%
	(5, 1, 2)	167594	2.242%
	(5, 0, 3)	17121	0.229%
9	N/A^	92	0.001%

* The structures of Ca ions are presented in parentheses as the number of Ca-O_s bonds, the number of ligand teeth, and the number of Ca-H_2O_w bonds.

^ There are no separate statistical structures for the nine-coordinated Ca ions due to the extremely small proportion.

Supplementary Table 4. The MD model parameters of β-C_2S (100) and M3-C_3S (010).

Type	a (Å)	b (Å)	c (Å)*	α (°)	β (°)	γ (°)
β-C_2S (100)	27.03	37.21	259.93	90	90	90
M3-C_3S (010)	34.60	27.84	292.05	90	90	70.54

* The difference between β-C_2S (100) and M3-C_3S (010) thicknesses was due to the selection of the same period during surface model preparation and the preservation of the periodicity of the in-plane crystals.

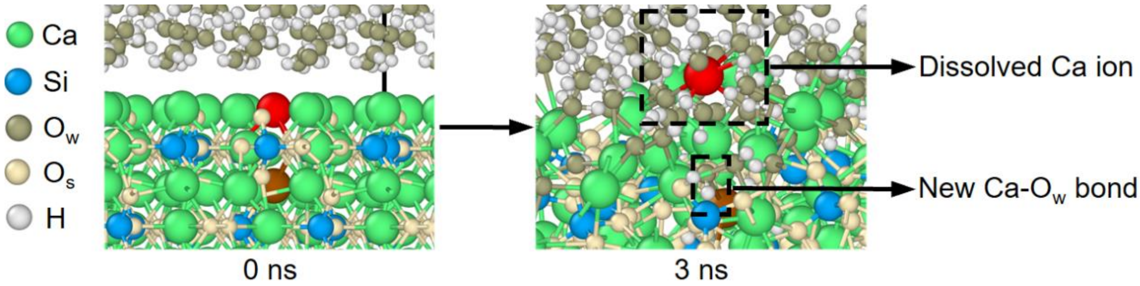

Supplementary Figure 1. The snapshots of Ca ions in M3-C_3S (010) at 0 ns and 3 ns. The Ca ions marked by red and brown color indicate the upper and lower Ca ions, respectively.

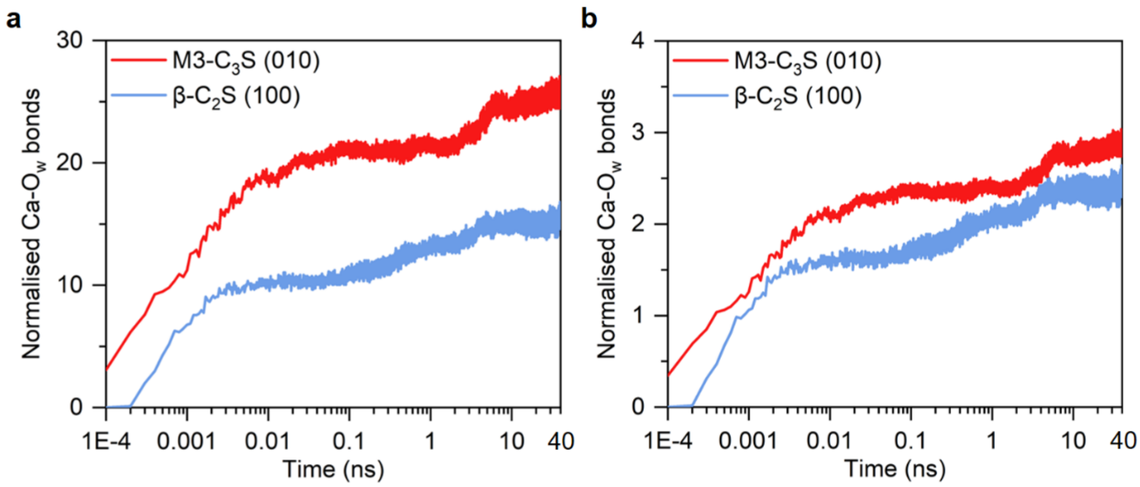

Supplementary Figure 2. The number of normalised Ca-O_w bonds of M3-C_3S (010) and β-C_2S (100). a Normalised Ca-O_w bonds with respect to the initial surface area. **b** Normalised Ca-O_w bonds with respect to initial surface calcium density. Note that a calcium atom was considered to be a surface calcium atom when it was exposed to the vacuum. The total number of initial surface calcium atoms from M3-C_3S (010) and β-C_2S (100) were 81 and 64, respectively.

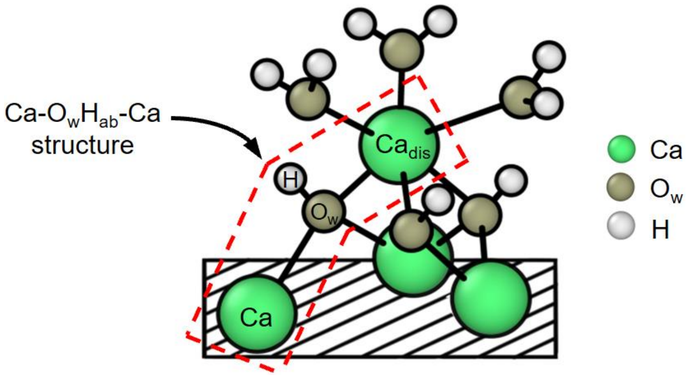

Supplementary Figure 3. The Ca-$O_w$$H_{ab}$-Ca structures of the dissolved Ca ion. The dissolved Ca ion consists of three Ca_{dis}-O_wH (tridentate) bonds and three Ca_{dis}-H_2O_w bonds.

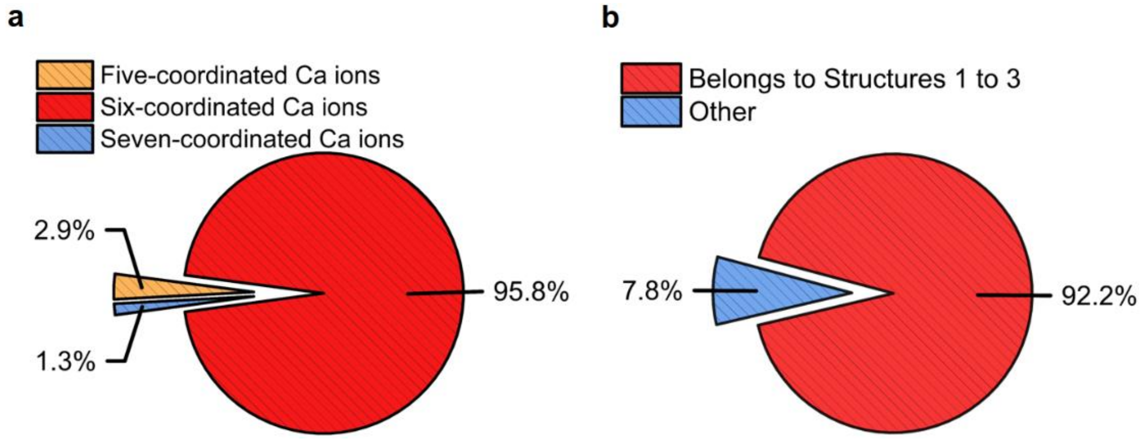

Supplementary Figure 4. The percentage of dissolved Ca ions in M3-C_3S (010) that appeared within 40 ns of the initial hydration process. a The coordination number of the dissolved Ca ions. **b** The structures of the dissolved six-coordinated Ca ions.

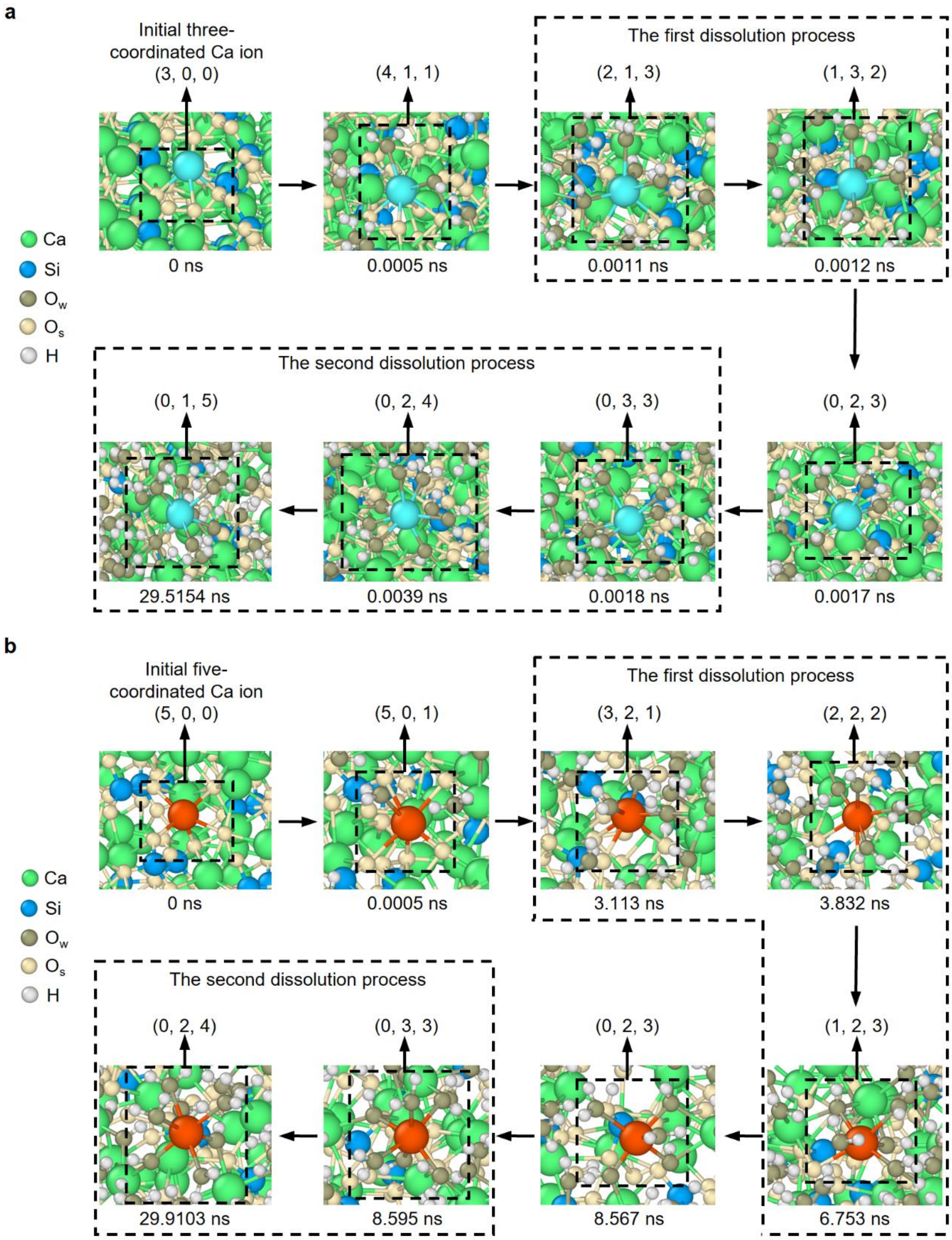

Supplementary Figure 5. The snapshots of dissolution pathways of Ca ions with different initial coordination numbers in M3-C_3S (010). a The initial three-coordinated Ca ions. **b** The initial five-coordinated Ca ions. The initial three- and five-coordinated Ca ions are marked by blue and red color, respectively. Some of the free

water molecules are removed in the snapshots to provide a clearer view of the Ca ion structures. The structures of Ca ions are presented in parentheses as the number of Ca-O_s bonds, the number of ligand teeth, and the number of Ca-H_2O_w bonds.

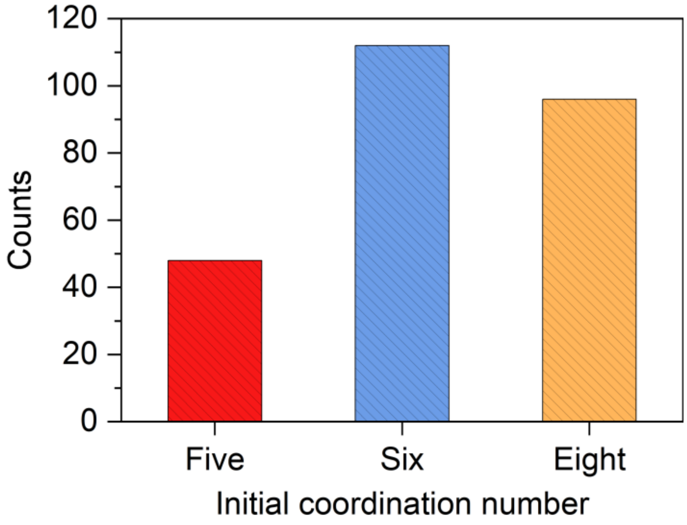

Supplementary Figure 6. The number of Ca ions with different initial coordination number in β-C_2S (100).

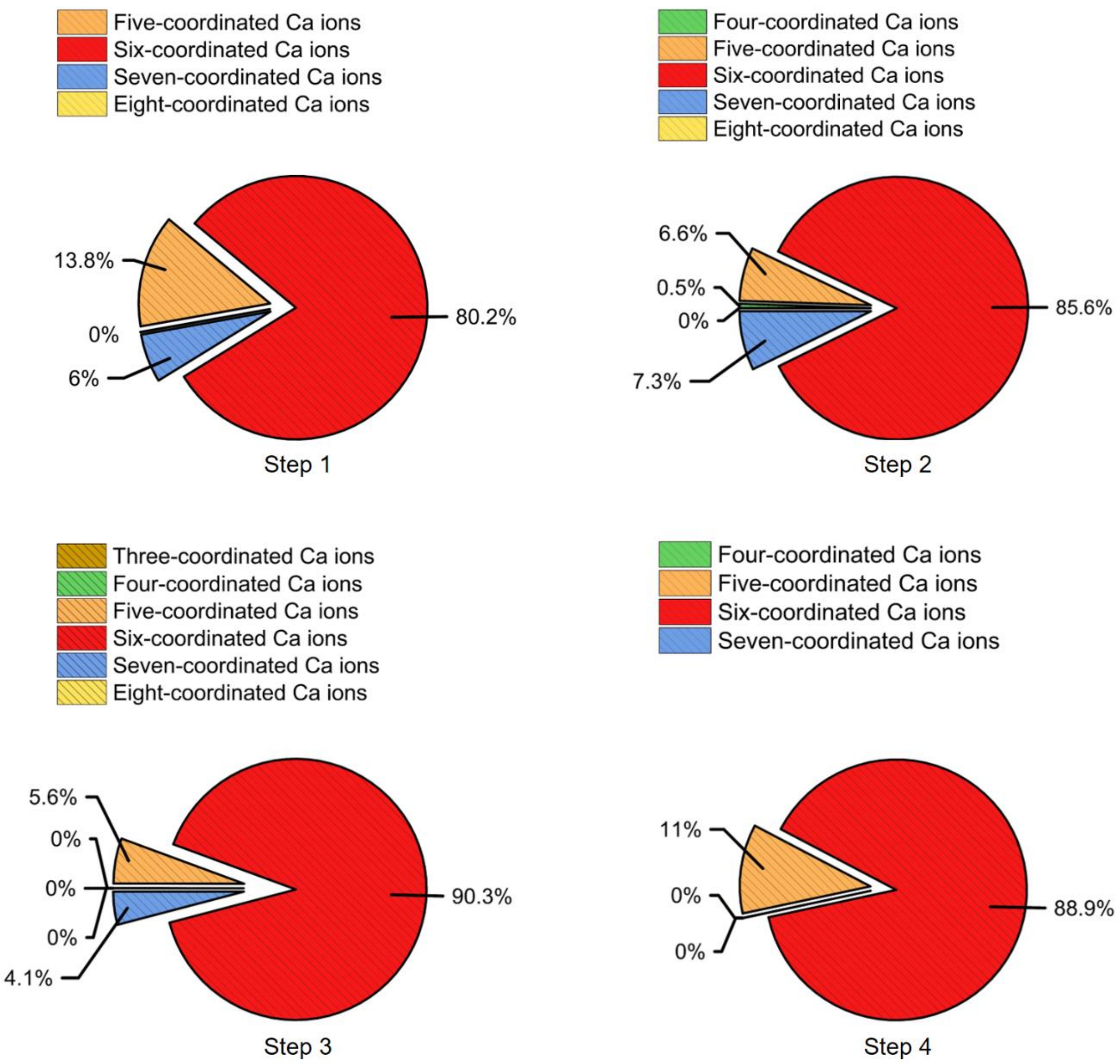

Supplementary Figure 7. The percentage of Ca ions with different coordination numbers in Steps 1 to 4 of the β-C_2S (100). The 0% percentage indicates that the number is extremely small.

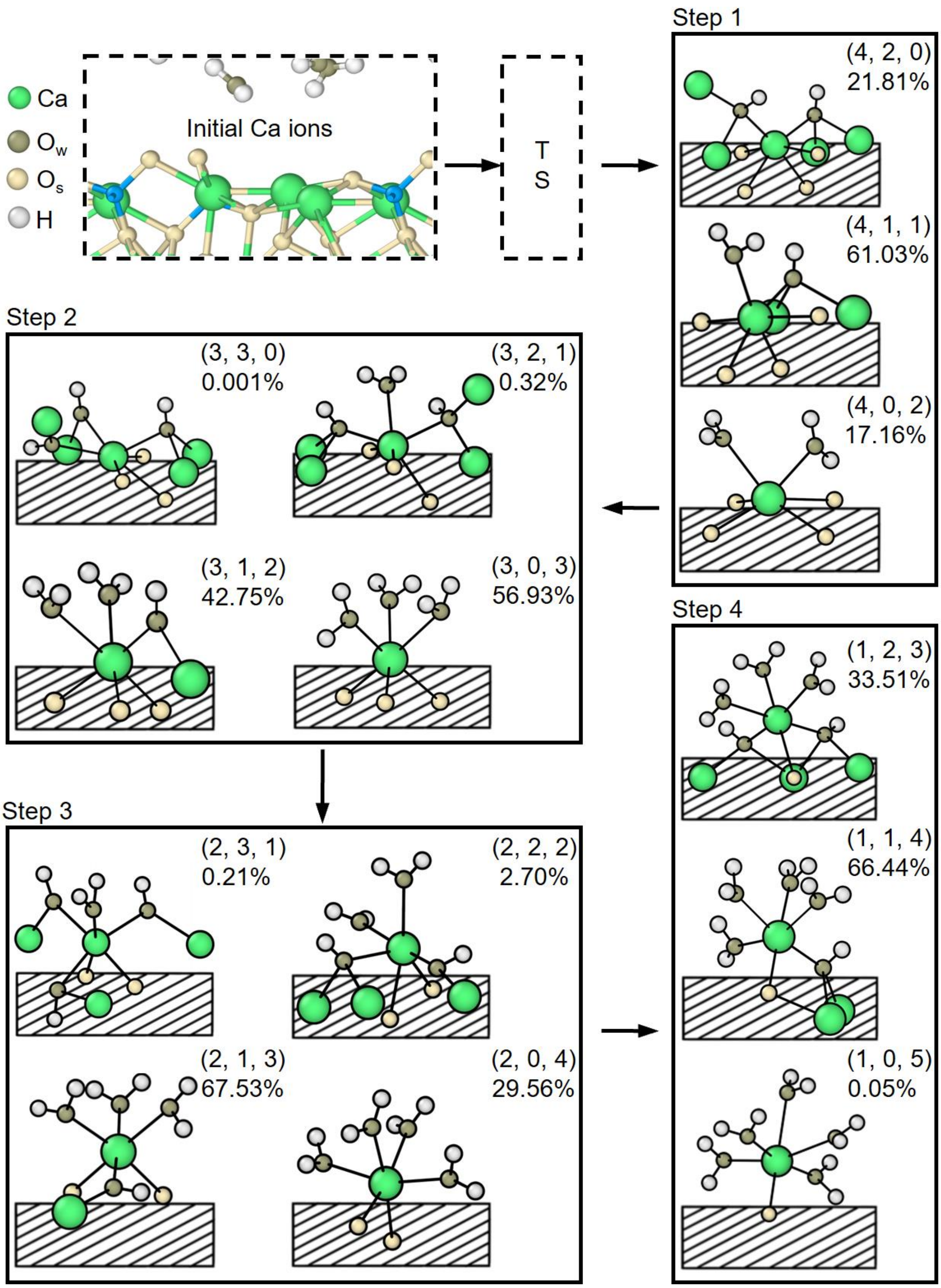

Supplementary Figure 8. The conceptualized flowchart of the Ca ions dissolution in β-C_2S (100). The structures of Ca ions are presented in parentheses as the number of

Ca-O_s bonds, the number of ligand teeth, and the number of Ca-H_2O_w bonds. The surface is shown as the shaded box.

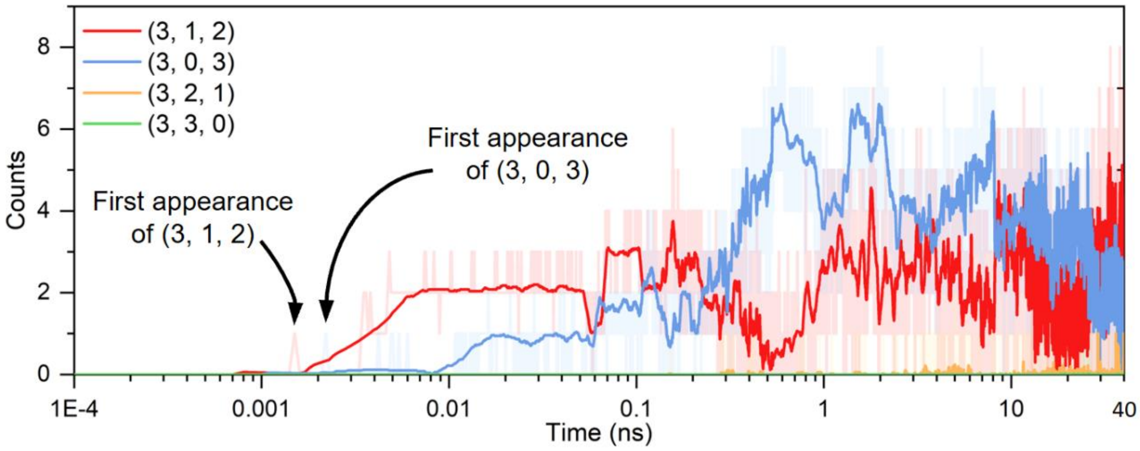

Supplementary Figure 9. The evolution of Ca ions' structures of Step 2 in β-C_2S (100) with time. The structures of Ca ions are presented in parentheses as the number of Ca-O_s bonds, the number of ligand teeth, and the number of Ca-H_2O_w bonds. The raw data curves in Supplementary Figure 9 are partially transparent and the smoothed curves are highlighted. The complete data of the raw and smoothed curves is provided in the Source Data file.

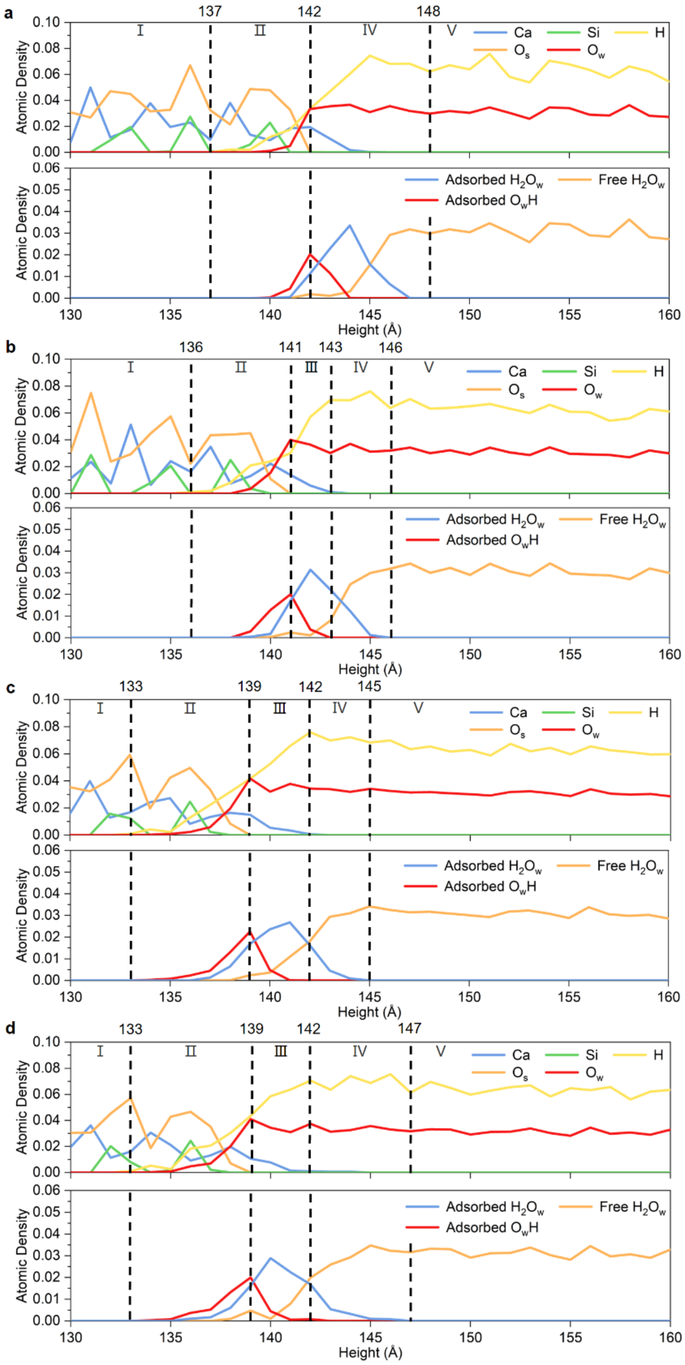

Supplementary Figure 10. The solid/water interface of the initial hydration process with time. The atomic and structure density of M3-C_3S (010) at **a** 0.1 ns, **b** 1.5ns, **c** 6 ns, and **d** 40 ns. The density is averaged 1 ps before different timelines to avoid excessive volatility.

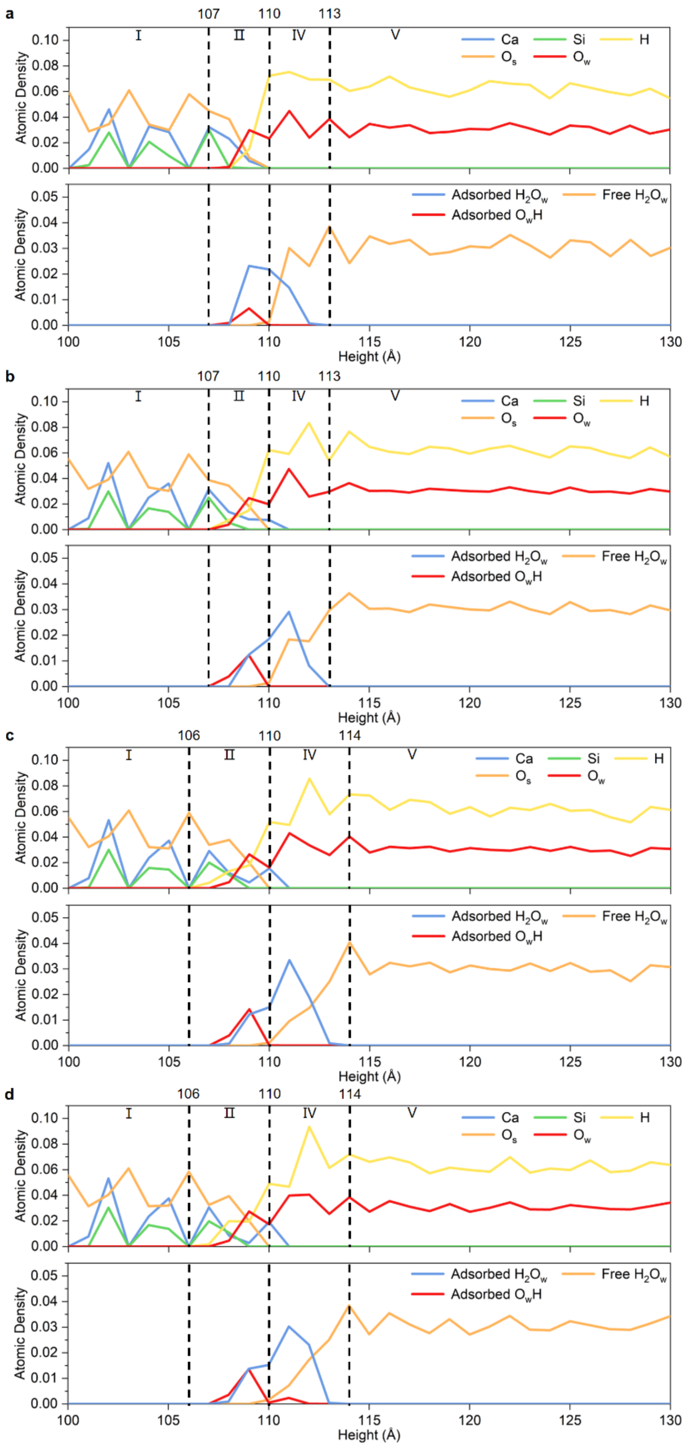

Supplementary Figure 11. The solid/water interface of the initial hydration process with time. The atomic and structure density of β-C_2S (100) at **a** 0.1 ns, **b** 1.5ns, **c** 6 ns, and **d** 40 ns. The density is averaged 1 ps before different timelines to avoid excessive volatility.

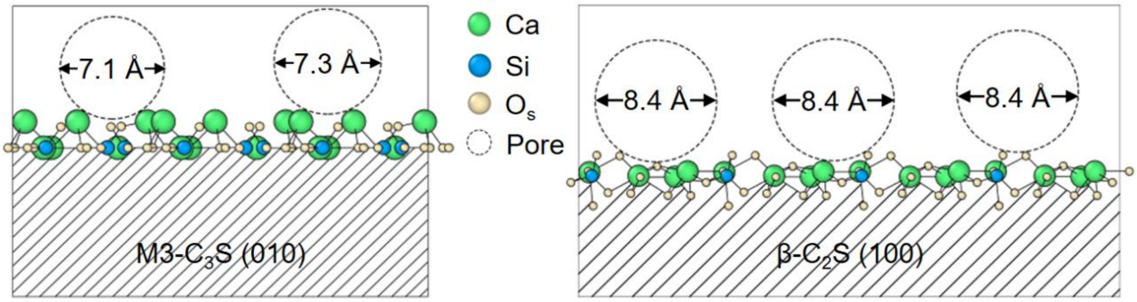

Supplementary Figure 12. The schematic figures of the large pore distribution of M3-C_3S (010) and β-C_2S (100) at 0 ns.

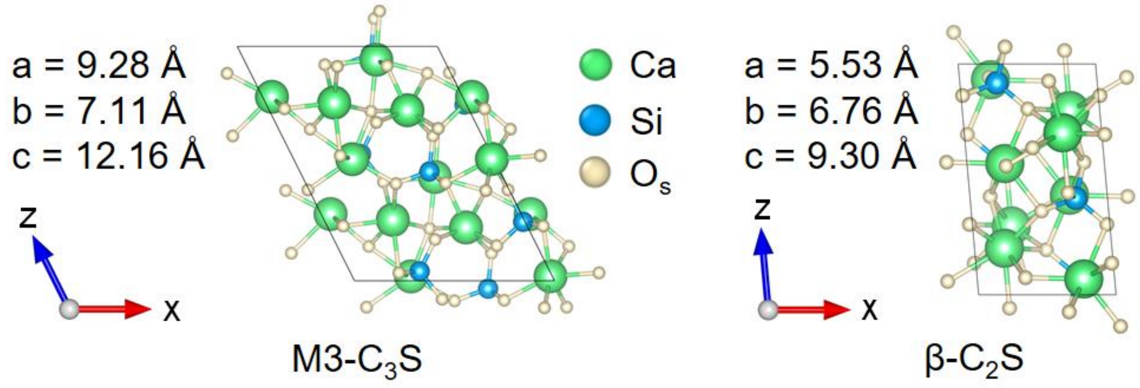

Supplementary Figure 13. The optimized unit cell structures of M3-C_3S and β-C_2S.

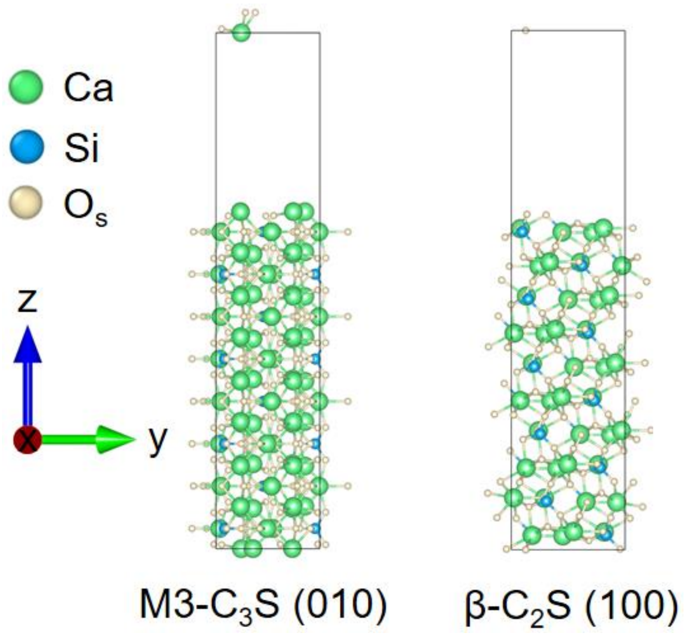

Supplementary Figure 14. The cleaved surfaces of M3-C_3S (010) and β-C_2S (100).

Supplementary Note 1

The 1.9 times is a comparison between the incremental values within 1.5–6 ns. The number of bonds at 1.5 ns and 6 ns is provided in the Supplementary information (Supplementary Table 1). The overall Ca-O_w and O_w-H_{ab} ratios for β-C_2S (100) are 1.19 and 1.28 at 1.5 and 6 ns, respectively. The overall ratios for M3-C_3S (010) are lower, 1.04 at both 1.5 and 6 ns (Supplementary Table 1).

Moreover, the structures of six-coordinated Ca ions of β-C_2S (100) between 1.5 and 6 ns are counted (Supplementary Table 2). The Ca ions with ligand teeth are recorded 77075 times (68.62%) and the Ca ions without ligand teeth are recorded 35251 times (31.38%). Therefore, it is believed that ligand teeth are widely present in the dissolution process of β-C_2S (100) between 1.5 and 6 ns.

Supplementary Note 2

The initial pore size is contributed by the vacuum layer of the pore model (Supplementary Fig. 11). The vertical coordinate of Fig. 5c and Fig 5d is the derivative distribution of the pore size. The value of the vertical coordinate of β-C_2S (100) is larger than that of M3-C_3S (010), indicating that the β-C_2S (100) pore diameter is concentrated between 8 and 9 Å. The surface atoms of β-C_2S (100) are more uniformly arranged than that of M3-C_3S (010).

Moreover, since the large diameter pores are mainly contributed by the vacuum layer, we are more interested in the formation of small diameter pores, since these pores are beneficial to proton transfers in the near-surface region.

Supplementary Note 3

The first comparison is made with the Li et al.[1], in which the Ca dissolution process of M3-C_3S was initially discussed. The model in the literature contained less than 1000 atoms. The simulation time remained on the scale of ps, approximately at 0.01 ns. The metadynamics approach was used in the literature to accelerate the structural evolution. In this study, we do not use any accelerated dissolution method. This means that a large number of chemical reactions have occurred on the surface before Ca ions dissolution and amorphous products are continuously formed on the surface. This will provide a realistic interface and reaction path for cement hydration, which is essential for the comprehensive analysis of the cement hydration mechanism.

The second comparison is made with the study written by the authors in 2015[2], ACS Appl. Mater. Interfaces 2015, 7, 27, 14726-14733. The ACS study used the molecular dynamics of reaction force fields to understand the hydration of tricalcium silicate. First, static characteristics such as surface energy and water adsorption energy were analyzed. This led to the conclusion that a dynamic study of mineral hydration is necessary to observe local chemical reactions and reveal the hydration process. However, limited by the time scale (2 ns), it remained at the stage of water molecule adsorption, including the formation of hydroxyl pairs and the hopping of H ions to the interior, and no dissolution of Ca ions was observed. Therefore, the ACS paper could not shed light on the Ca dissolution, which is the major contribution of this study. Moreover, the

simulation model is larger and a detailed comparison between M3-C_3S and β-C_2S has been presented in the current study.

Supplementary Note 4

This study provides four main contributions to the initial cement hydration, namely a new Ca dissolution process, two new general dissolution pathways, a key structure for Ca ions dissolution, and a detailed characterization of the hydration process.

A new Ca dissolution process: For M3-C_3S in previous studies, it was considered that Ca ions dissolve directly after breaking all bonds with surface O ions, which is the dissolution of Ca ions by definition. However, the results show that the complete detachment of Ca ions from surface O ions in M3-C_3S does not achieve free movement in the water layer, but enters a new dissolution process, namely the second dissolution process. The second dissolution process requires a tens of thousands fold longer period than the first dissolution process. The discovery of this new process revises our current understanding of Ca ions dissolution in M3-C_3S and provides a benchmark knowledge for the dissolution mechanism of other silicate minerals.

Two new general dissolution pathways: In this study, the general pathways of Ca ions dissolution in M3-C_3S and β-C_2S have clarified after extensive statistics analysis, which is a breakthrough in Ca dissolution. The authors consider the revealed Ca ions dissolution pathways to be more realistic and representative since they are obtained from non-speculative, highly-accurate, and non-accelerated structural evolution MD simulations. That marks the opening of a new chapter in the study of initial cement hydration, from the stage of water molecules adsorption to the stage of Ca dissolution.

The general Ca dissolution pathway will be an important theoretical basis for the Ca dissolution stage and a key step toward exploring the nucleation of cement hydration.

A key structure for Ca dissolution: In this study, a new ligand teeth structure is observed, which is specifically expressed as Ca-O_wH_{ab}-Ca. The ligand teeth widely exist in the Ca ions dissolution of M3-C_3S and β-C_2S, the appearance of which facilitates the detachment of Ca ions from the surface O ions. The formation of more ligand teeth structures is observed during the Ca ions dissolution of M3-C_3S. Moreover, multiple ligand teeth structures are also observed in the intermediate layer of C-S-H structure, and such ligand teeth structures will play an important role in hydrates formation.

A detailed characterization of the hydration process: The initial hydration process of the cement at 40 ns is characterized in multiple ways. These characterizations also led to many new findings that are not previously found, for example, the appearance of aqueous layers and the small pores favoring H transfer in M3-C_3S. These characterization results demonstrate in great detail the differences in the early hydration of M3-C_3S and β-C_2S. Moreover, this study provides a huge simulation data for the initial cement hydration process, which paves the way for further in-depth as well as multi-faceted studies of cement hydration in the future.

Supplementary references

1. Li, Y., Pan, H., Liu, Q., Ming, X. & Li, Z. Ab initio mechanism revealing for tricalcium silicate dissolution. *Nat. Commun.* **13**, 1253 (2022).

2. Manzano, H., Durgun, E., López-Arbeloa, I. & Grossman, J. C. Insight on Tricalcium Silicate Hydration and Dissolution Mechanism from Molecular Simulations. *ACS Appl. Mater* **7**, 14726-14733 (2015).
